# Differential Proteome Analysis of Extracellular Vesicles from Breast Cancer Cell Lines by Chaperone Affinity Enrichment

**DOI:** 10.3390/proteomes5040025

**Published:** 2017-10-08

**Authors:** Steven G. Griffiths, Michelle T. Cormier, Aled Clayton, Alan A. Doucette

**Affiliations:** 1 Minervagen Biotechnologies Corporation, Tucson, AZ 85704, USA; stevegriffiths@x0s0me.com; 2 Atlantic Cancer Research Institute, Moncton, NB E1C8X3, Canada; caissiemichelle@hotmail.com; 3 School of Medicine, Cardiff University, Wales, CF14 4XN, UK; claytona@cardiff.ac.uk; 4 Department of Chemistry, Dalhousie University, 6274 Coburg Road, Halifax, NS B3H 4R2, Canada

**Keywords:** extracellular vesicles, breast cancer cell lines, Vn96, heat shock proteins, glycolysis, GELFrEE

## Abstract

The complexity of human tissue fluid precludes timely identification of cancer biomarkers by immunoassay or mass spectrometry. An increasingly attractive strategy is to primarily enrich extracellular vesicles (EVs) released from cancer cells in an accelerated manner compared to normal cells. The Vn96 peptide was herein employed to recover a subset of EVs released into the media from cellular models of breast cancer. Vn96 has affinity for heat shock proteins (HSPs) decorating the surface of EVs. Reflecting their cells of origin, cancer EVs displayed discrete differences from those of normal phenotype. GELFrEE LC/MS identified an extensive proteome from all three sources of EVs, the vast majority having been previously reported in the ExoCarta database. Pathway analysis of the Vn96-affinity proteome unequivocally distinguished EVs from tumorigenic cell lines (SKBR3 and MCF-7) relative to a non-tumorigenic source (MCF-10a), particularly with regard to altered metabolic enzymes, signaling, and chaperone proteins. The protein data sets provide valuable information from material shed by cultured cells. It is probable that a vast amount of biomarker identities may be collected from established and primary cell cultures using the approaches described here.

## 1. Introduction

According to the American Cancer Society, breast cancer remains the most commonly diagnosed cancer among women in the United States, predicting over 250,000 new cases in 2017 and accounting for 30% of all new cancer diagnoses [1]. Breast cancer further ranks second, behind lung cancer, in terms of cancer cell death among women with >40,000 incidences in 2017. While five-year breast cancer survival rates approach 100% in the case of localized tumors (stage 0 or I), the prognosis for metastasized (stage IV) breast cancer is poor, with only 22% survival at 5 years [2]. Early detection of breast cancer patients is thus crucial to improving recurrence-free survival and quality of life. Markers that distinguish invasive breast cancer phenotypes are urgently required.

Conventional screening tools, such as self-examination, mammography, diagnostic imaging (ultrasound, MRI), genetic screens (e.g., BRCA1), and tissue biopsies [3,4], collectively present vital tools for breast cancer detection. As with all tests, limits in sensitivity and specificity will miss some cancers (false-negatives); in other cases, abnormal findings associated with benign disease (false positives) will direct between 55% and 75% of women into unnecessary and potentially toxic chemotherapy [5,6,7]. The next generation of diagnostic and prognostic tests is continuously being sought. In particular, considerable effort is currently devoted to uncovering early molecular indicators released into the blood by cancer cells [8]. To this end, both DNA from circulating tumor cells [9] and microRNA released to plasma by cancer cells [10] have been reported as potential biomarkers. Differentially expressed protein biomarkers have also been observed in plasma [11]. The presence of high abundance background plasma proteins challenges the detection of low abundance proteins of interest, pointing to a need for more selective and sensitive indicators of breast cancer. 

Exosomes are a subclass of extracellular vesicles (EVs), constituting membranous spheroids between 30 and 200 nM released from cells. Initially regarded as “cell dust”, EVs are now unequivocally associated with various types of cancer, and carry specific profiles of proteins, nucleic acids, and lipids that serve to transmit information to other cells. Once secreted by cells into the extracellular medium, EVs retain features of the originating tissue environment [12]. EVs have also been shown to alter the metabolic activity of neighboring cells [13], and thus, may have important functional implications for tumor growth and differentiation. Not only are EVs with specific molecular payloads released by cancer cells, but they are secreted in relative higher abundance. Hence, protein profiling of cancer cell-derived exosomes has gained attention as a favorable source of protein biomarkers. Multiple approaches are available to isolate exosomes from the bulk sample. The most common approach is differential ultracentrifugation (UC); although laborious and producing a lower yield, UC remains the “gold standard” in obtaining exosomes in high purity [14]. Whilst differential centrifugation remains the laboratory method of choice [15], it is an inefficient and poorly reproducible tool that is unsuited for handling even modest numbers of clinical samples [16,17]. Access to ultracentrifugation is also limited in most clinical diagnostic laboratories, demanding robust alternative EV preparations for future translational utility. Alternative approaches, including dialysis, size exclusion chromatography, and polymer- or antibody-induced precipitation, are available [14]. 

In an alternative strategy, a synthetic peptide (Vn96) with high affinity for heat shock proteins (HSPs) has recently been shown to selectively isolate EVs by co-precipitation [18]. Not surprisingly, HSPs are overexpressed in cancer cells [19], where maintenance of protein homeostasis is at a premium, due to hypoxia, low pH, and limited glucose availability. HSPs are also found in high abundance on the plasma membrane surface of exosomes [20], thus, the Vn96 pulldown represents an ideal strategy to enrich EVs shed from cancer cells. The Vn96 affinity peptide has previously been shown to recover EV material from urine, plasma, as well as cancer cell lines [21,22,23].

In this study, we demonstrate for the first time the utility of Vn96 in isolating breast cancer EVs for functional proteomic analysis of malignant phenotypes. We employ various breast cancer cells, including the adenocarcinoma models SKBR3, an invasive HER2^+^ cell and MCF-7, a luminal A cell line model. MCF-10a, a non-tumorigenic breast fibrocystic disease model, is also employed as a control. In-depth proteome analysis by LC-MS/MS, following GELFrEE fractionation of exosomal proteins, reveals numerous differentially expressed proteins, which readily distinguish the various cell lines. The proteins convey unique profiles, particularly in terms of their metabolic and chaperone activity, providing insight into the biological function of breast cancer-derived EVs and potentially pointing a strategy to uncover protein biomarkers for early breast cancer detection. 

## 2. Materials and Methods

### 2.1. Cell Culture and Sample Collection

Breast cancer cell lines (SKBR3, MCF-7), along with human breast epithelial cell MCF-10a, were obtained from ATCC, and cultured according to recommended guidelines using CELLiNE Integra bioreactor flasks (Sigma, Oakville, ON, Canada), which permits addition of fresh media to layered and non-adhesive cells maintained on the opposing side of a 10 kDa membrane. The media was supplemented with exosome-free fetal bovine serum (FBS), prepared by centrifugation of the FBS (100,000*g*, 2 h, 4 °C) prior to use. CELLiNE media was harvested at 7–8 weeks, as optimized for the collection of exosomes [24], and immediately combined with 5 µL protease cocktail inhibitor III per mL (Sigma). Remaining cells in the media were cleared by centrifugation (1000*g*, 10 min) and microparticulate matter was further removed by centrifugation (17,000*g*, 5 min), followed by syringe filtration through a 0.22 µm membrane. Filtered solutions were stored at 4 °C. 

### 2.2. Ultracentrifugation and Sucrose Density Gradient Fractionation

The filtered SKBR3 cellular media was subject to 45 min ultracentrifugation (200,000*g*, 4 °C). The pellet was resuspended in 100 µL PBS, and overlaid onto a sucrose gradient (0.2–2.5 M), then spun for an additional 1 h (100,000*g*). A total of 11 fractions were harvested, and the refractive index was determined. Each density fraction was split in two equal portions of 250 µL, with one subject to Vn96 affinity pull-down while the other served as a control. 

### 2.3. Vn96 Affinity Capture of EVs

Peptide Vn96 (New England Peptide, Gardener, MA, USA) was used to precipitate vesicular material from the sucrose density fractions (Section 2.2), or from 1.9 mL of the microparticle-free breast cancer cell media by adding 2.5 μL (or 10 μL for the cell culture media) of a 10 μg/μL stock solution prepared in Extraction Buffer I of the subcellular proteome extraction kit (S-PEK, Millipore Sigma), and also containing 0.04% sodium azide. The solution was briefly vortexed, and incubated overnight at 4 °C. Complexed material was pelleted by centrifugation (3000*g*, 5 min, room temperature). The supernatant was removed, and the pelleted material was subject to two washes with 1.9 mL PBS with 10 μL protease inhibitor, followed by centrifugation as above. The washed pelleted complex was visible as a translucent straw-yellow residue. 

### 2.4. EV Purity by Immunobloting and Transmission Electron Microscopy

Vn96-captured EVs were resuspended by vortex in SDS-PAGE sample buffer and heating at 95 °C for 5 min. Twenty microliter volumes were resolved on BioRad Criterion gels using XT-MES or XT-MOPS electrophoresis running buffers (BioRad, Hercules, CA, USA). Resolved proteins were transferred to either supported nitrocellulose (BioRad) or PVDF (Millipore) using standard procedures. Total protein on blots was visualized with reversible stain using either the MemCode kit (PIERCE) for nitrocellulose, or Red Alert Ponceau S (EMD Chemicals Gibbstown NJ). Membranes were blocked in PBS containing 5% skimmed milk and 0.1% Tween-20 for 1 h, then incubated overnight at 4 °C in primary antibody solutions (1:1000 dilution, prepared in blocking buffer with exception of 3% milk powder). Four 10 min washes followed (blocking buffer without milk powder), then the blot was incubated for 30 min at room temperature in secondary antibody (1:2000 dilution, HRP-labelled antibody to the Ig consistent with primary antibody). All antibodies were obtained from Santa Cruz Biotechnology. HRP signal was produced using SuperSignal West Dura substrate (Pierce). The chemiluminescent image was captured using the ChemiGenius system (Syngene, Cambridge, UK). 

For transmission electron microscopy, Vn96-captured EVs were prepared by fixation in aldehydes and osmium tetroxide [21]. The fixed pellet was embedded in epoxy resin and prepared as 50 µm sections. The sections were processed and examined by standard transmission electron microscopy.

### 2.5. Proteome Analysis

The Vn96 pellet was resuspended in 250 µL of SDS-PAGE sample buffer, supplemented to a final concentration of 4 M urea and 25 mM TCEP reducing agent (Pierce). Following heating (95 °C, 10 min), 150 μL suspended protein solutions were respectively loaded onto each of three GELFrEE cartridges (8%, 10%, and 12% Tris Acetate, Expedeon, San Diego, CA, USA) and resolved according to the manufacturer’s operating guidelines. With each run, 12 fractions were collected as 150 μL aliquots. Together with non-fractionated Vn96 proteome pellets, a 7 µL portion of each collected GELFrEE fraction was subject to SDS-PAGE and silver staining, for visualization of mass-based separation and recovery per fraction.

Bottom up proteomic analysis of the GELFrEE-fractionated proteome first proceeded via SDS removal through chloroform–methanol–water precipitation, as described previously [25]. The resulting protein pellet was resolubilized in 20 µL of 8 M urea, then diluted to a final volume of 100 µL in 50 mM Tris buffer (pH 8). Proteins were reduced following addition of 5 µL of 200 mM DTT (30 min, 55 °C), then alkylated by adding 10 µL 200 mM iodoacetamide (30 min, room temperature, dark). Proteins were digested overnight at 37 °C following addition of 1 µg trypsin per fraction, and the reaction was terminated with 10 µL of 10% TFA. 

Mass spectrometry was on an LTQ classic linear ion trap (ThermoFisher, San Jose, CA, USA), coupled to an Agilent 1200 HPLC system. Digested protein fractions were desalted by offline reversed phase HPLC with UV detection [26], loading one-third of the total volume of purified protein onto a 75 µm × 30 cm self-packed C12 column (3 µm Jupiter beads, Phenomenex, Torrance, CA, USA). Peptides were separated using a 1 h linear solvent gradient from 5% acetonitrile/water/0.1% formic acid to 35% acetonitrile/water/0.1% formic acid at flow rate of 0.25 µL/min. The LTQ operated in data dependent mode (MS followed by zoom scan and tandem MS of the top three ions), with 30 s dynamic exclusion over a mass range covering the full isotopic distribution of the peptide.

Peptide identification used the Thermo Proteome Discoverer (v. 1.3, ThermoFisher, Mississauga, Canada) software package and the SEQUEST searching algorithm. MS spectra were searched against the human UniProt database, at a mass tolerance of 1 Da, allowing static cysteine carbamidomethylation and dynamic oxidation of methionine, and up to two missed cleavages per peptide. A peptide false discovery rate of 1% by decoy database searching, and minimum of one unique peptide per protein were employed for data filtering. Relative protein abundance was obtained via spectral counting [27] with normalized peptide spectral matches (PSMs), obtained by way of a ratio of the total number of PSMs observed in the SKBR3 cell line (highest PSM total) to that of the total PSMs from the given cell line. Functional annotation was performed by Ingenuity Pathway Analysis (QIAGEN Bioinformatics, Redwood City, CA, USA). 

## 3. Results and Discussion

We report a comparative proteome investigation on an in vitro model of breast cancer, examining the extracellular media from SKBR-3 (invasive cancerous cell), MCF-7 (non-invasive), and MCF-10a (immortal but non-cancerous cells). While cells adapted to grow in plastic flasks are regarded as very different from those obtained in vivo, material secreted by cancer cells into the external environment in vitro is likely to produce a similar proteomic profile, reflecting the original growth from which it was derived [28]. The Vn96 peptide was employed to selectively capture EV material released by cultured cell lines into their growth media. Vn96 targets heat shock proteins (HSPs), overexpressed on the surface of aggressive cancer cells, and by extension, their derivative vesicles [29]. The Vn96 peptide has been shown to capture exosome-like vesicles containing proteins comparable to EV preparations by traditional ultracentrifugation when analyzed by Western blot [21].

**Figure 1:**
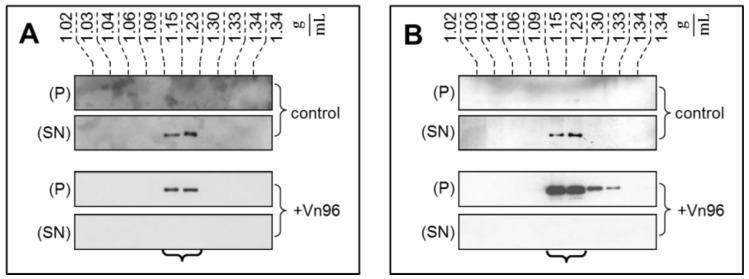
Western blot analysis of fractions recovered from sucrose density gradient isolation of SKBR3 extracellular vesicles (EVs) reveals the presence of exosomal marker proteins TSG101 (**A**) and Alix (**B**). These proteins remain in the supernatant in the control sample following low speed centrifugation. With addition of Vn96, the low speed spin recovers the same proteins in the pelleted material.

To demonstrate the specificity of Vn96 to pellet EV material, exosomes from SKBR3 were harvested through conventional ultracentrifugation with separation into characteristic flotation zones by sucrose density fractionation. As shown in Figure 1, the exosomal marker proteins TSG101 and Alix were isolated in the fractions from 1.15 to 1.23 g/mL. Moreover, while these marker proteins remain suspended in the supernatant (SN) following low speed centrifugation, the addition of Vn96 resulted in recovery of exosomal markers in the pellet (P) of the low speed spin. These observations demonstrate the capacity of Vn96 to concentrate proteins associated with vesicular material.

**Figure 2:**
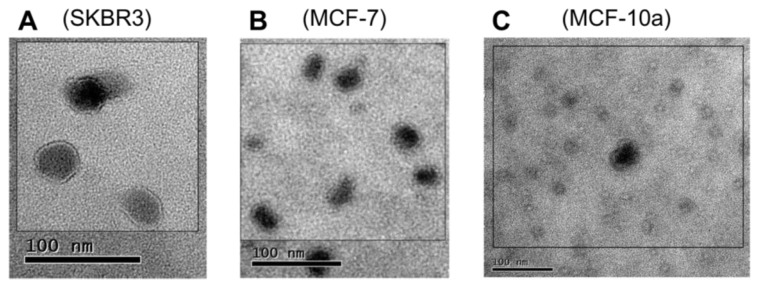
TEM images showing the distribution of microvesicular bodies recovered from the extracellular media by precipitation with Vn96 peptide of (**A**) SKBR3; (**B**) MCF-7; (**C**) MCF-10a. The size distribution of the particles is consistent with small extracellular vesicles.

The Vn96 peptide was next employed to directly recover EV materials from filtered bioreactor cell culture media. When observed by TEM Figure 2, material pulled down by Vn96 generally consisted of bilayer orb structures between 30 and 50 nm in diameter, with electron dense centers. Such features were also evident in the MCF-10a cell line, indicative that HSP-decorated vesicles are also released by these cells. In recent years, the term “small EVs” has been used as an alternative to exosomes [30]. Vn96 may capture a subset of small EVs, as defined by surface accessorization of HSPs.

**Figure 3:**
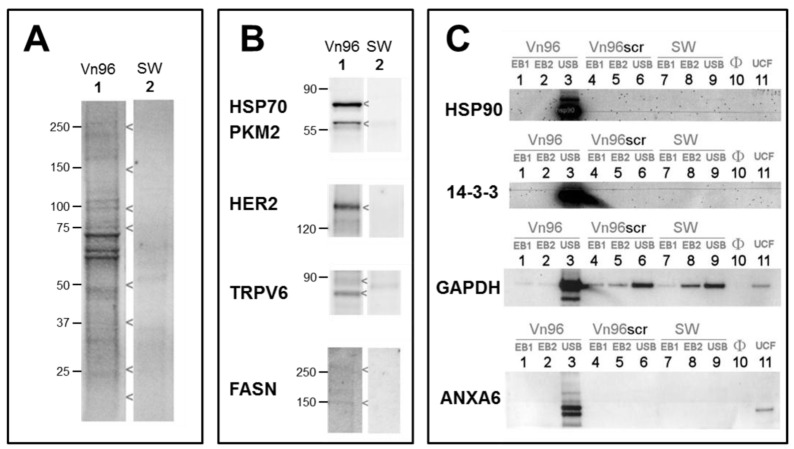
Immunoblots probing for exosomal marker proteins in the Vn96 pellets isolated from SKBR3 cell culture media. (**A**) A random peptide (SW) precipitates few proteins from the extracellular media, as evident from a total protein stain; (**B**) Marker proteins are observed in the immunoblots of the Vn96 pulldown following solubilization in SDS-containing buffer (USB); (**C**) Extracting the Vn96 pellet with mild detergent buffers, EB1 & EB2, from Millipore’s subcellular proteome extraction kit, S-PEK, will not release proteins from the Vn96 pellet, permitting incorporation of a wash step to improve purity. As controls, equivalent volumes of culture medium were processed by ultracentrifugation (UCF), the pellet was resuspended in electrophoresis buffer and loaded directly (Lane 11).

Electrophoretic profiling of Vn96 pull-downs yielded robust total protein profiles (e.g., from SKBR3, Figure 3A lane 1). Replacement of Vn96 with a random peptide (SW) yielded faint profiles likely to be aggregates of albumin and immunoglobulin from culture medium (Figure 3A, lane 2). The heat shock proteins (HSP70, HSP90) targeted by Vn96 were depleted from the medium and exclusively recovered in the low centrifuge speed pellet. Employing a random peptide (SW), or scrambled amino acid sequence of Vn96 did not pellet these proteins (Figure 3B). Western blots of EV material also identified PKM2, receptor kinase HER2, membrane protein TRPV6, and fatty acid synthase; none of which were recovered using the null peptide (Figure 3B). The stability of EVs isolated by Vn96 from SKBR3 is also evident in Figure 3C, which employs various commercial detergent washes (components of Millipore’s subcellular proteome extraction kit, S-PEK). As seen in lanes 1 and 2 of Figure 3C, neither cytosolic Extraction Buffer I (EB1), which contains the mild detergent digitonin, nor membrane and organelle Extraction Buffer II (EB2), containing Triton X-100, would solubilize the EV marker proteins from the Vn96 pellet. Similar results are obtained from the other cell lines. Though EV marker proteins are retained following EB1 and EB2 washes, weakly associated proteins are liberated from the EV pellet by these buffers (see Supplementary Figure S1). These findings permit incorporation of stringent washes to further purify the EV fraction recovered in the Vn96 pellet. Immunoblot analysis on material recovered from the non-transformed MCF-10a cell culture media, as well as for MCF-7, with the breast cancer exosomal marker CD24 [31], illustrate that some, but not all, CD24 is released into the supernatant following incubation of the pellet with EB II (results not shown). Thus, we subsequently chose a PBS washing protocol, also under reducing conditions (25 mM TCEP), to achieve a balance of yield and purity. To fully solubilize EV proteins (lane 3 of Figure 3C), the pellet is boiled in SDS gel-loading buffer, supplemented with 4 M urea (USB). As a consequence, MS analysis demands an SDS-compatible proteomics workflow. 

**Figure 4:**
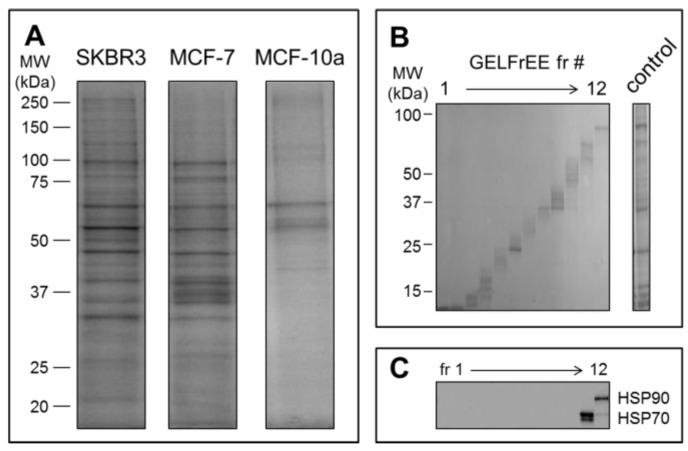
(**A**) SDS-PAGE of proteins recovered from Vn96 pellets reveal significantly more proteins from the cancerous cell lines SKBR3 and MCF-7 relative to the non-cancerous control MCF-10a; (**B**) GELFrEE fractionation resolves proteins according to molecular weight. Shown is the separation of SKBR-3 proteins from the EV pellet as obtained on the 8% (high molecular weight) gel cartridge; (**C**) Immunoblot of the identically resolved proteome as above, showing resolution of heat shock proteins (HSP70 and HSP90).

Mass spectrometry detection followed GELFrEE fractionation of the solubilized EV proteomes. GELFrEE enables recovery of proteins in an SDS-containing buffer, with separation according to molecular weight. SDS depletion via organic solvent precipitation permits LC/MS of trypsin-digested proteins. As demonstrated in Figure 4A, a higher abundance of EV proteins was recovered from the SKBR3 and MCF-7 cell lines, relative to the non-cancerous MCF-10a, in support of the theory that aggressive cancer cells will overexpress EV materials. The decreased abundance of proteins recovered by Vn96 from the MCF-10a cell media may also reflect a lower number of Vn96 binding opportunities, due to lack of surface expressed HSP/chaperones. Nonetheless, it is clear that a greater concentration of proteins is recovered from the tumorigenic cell lines. GELFrEE resolved the proteins over a mass range extending to ~100 kDa Figure 4B, isolating proteins in discrete fractions according to molecular weight (Figure 4C). A detailed listing of the identified proteins and peptides from each of the three cell lines are provided as supplementary files (Tables S1–S3).

**Figure 5:**
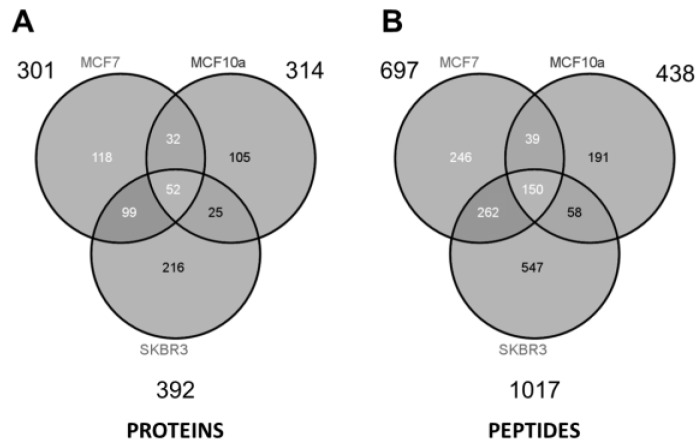
Venn diagrams summarize (**A**) the proteins and (**B**) peptides identified by mass spectrometry from each of the three cell lines. Detailed listings of identified proteins are provided as supplemental files.

As summarized in the Venn diagrams of Figure 5, some ~300 to 400 unique proteins were identified from each cell type (minimum 2 peptides per protein). The largest number of identified proteins (392) was seen from the most aggressive cancer cell line (SKBR3), and might be a reflection of the increased prominence of HSPs expressed on the EV surface. Considering all three samples and technical replicates (three GELFrEE runs per sample type), MS collectively identified 647 unique proteins across the various cell lines. Despite marked differences in protein concentration across the three cell lines, similar numbers of proteins were identified through MS. This may reflect on the limitations of an MS platform which favors detection of the most abundant components. While greater differences are reflected at the peptide level Figure 5, comparative analysis of the discrete proteomes is best presented through changes in the protein abundance. Here, spectral counting is employed as a means of assessing relative protein abundance across the three cell types. In total, we obtained 7400 peptide spectral matches (PSMs) from SKBR3; 4584 PSMs from MCF-7; and 2381 PSMs from MCF-10a, being indicative of the greater concentration of EV proteins recovered in the most invasive phenotype. Supplementary Table S4 details a full comparative assessment of the spectral matches observed per protein across the three cell lines. Supplementary Table S5 compares the identified proteins to the ExoCarta protein database [32], indicating that the majority of proteins detected (509 of 575 gene products) were previously found to be associated with exosomal material. 

Reflecting the origin of EVs as they are produced by the cell, and considering the cellular distribution of proteins identified from the EV fractions (Supplementary Figure S2), it is not surprising that several of the identified proteins were associated with the membrane (19%), or cellular surface (12%). The large number of extracellular proteins is reflective of the heat shock proteins along with other proteins normally released by cells. Table 1, Table 2 and Table 3 summarize the top 25 proteins (excluding probable contaminants), as categorized according to primary function (metabolism; chaperone and protein binding; skeletal and assembly). 

Multiple cytoskeleton proteins (keratins, actin, myosin, etc.) dominated the list of identified proteins (see Supplementary Tables S1–S3). Keratins are frequently encountered as unavoidable contaminant proteins during sample collection and processing. As such, suspected keratin contaminants (keratin type 1, 2, 5, 9, 14, 16) were among the identified proteins. These proteins exhibit minimal statistical difference in peptide spectral counts among the three cell types. However, such contaminants are easily distinguishable from bona fide cancer markers, such as cytokeratin 8, 18, and 19. These molecules are found at the surface of cancer cells, and are thus either incorporated as bystanders, or as part of the EV assembly and embarkation process. CK8 and CK18 are well documented as secreted cancer biomarkers, and may be detected in serum of patients with breast cancer receiving chemotherapy [33]. These markers were shown to resist extraction by wash buffers EB1 and EB2 (Supplementary Figure S1), and are thus likely to be strongly associated or embedded within the extracellular vesicles. CK19 has been suggested to be likely involved in driving the more aggressive tumor proliferation, invasion, and metastasis associated with HER-2/neu-positive tumors [34]. By their PSMs, each of these marker proteins were highly elevated in the cancer cell lines relative to MCF-10a (Table 1, Table 2 and Table 3). Thus, these proteins are included in the lists of relevant EV components. 

Considering the full list of proteins collectively identified in the EV material, a broad range of biological function is conveyed (Supplementary Figure S2). The largest group of proteins identified (18%) were associated with metabolic activity. The chaperone/binding proteins were also prominent among the identified groups. A preliminary comparison of the molecular pathways associated with the identified proteins is seen through Ingenuity Pathway Analysis (IPA), for which the top 12 pathways of each cell type are depicted in Supplementary Figure S3. The most distinguishing feature was the glycolysis/gluconeogenesis and the pentose phosphate pathways, represented at the top of IPA pathways represented in SKBR3 (second and fourth for MCF-7). By sharp contrast, metabolic enzymes constitutive of these pathways were essentially absent from MCF-10a. 

Cancers are dominated by metabolic pathways that vary from normative physiology with emphasis on accelerated uptake of glucose and glutamine, aerobic glycolysis, decreased mitochondrial activity, and enhanced lipogenesis. Table 4 provides a quantitative comparison of proteins involved in metabolic pathways. Perhaps the most well-known characteristic, the Warburg effect [35], refers to the avid consumption of glucose for direction into a glycolytic pathway with the accumulation of lactate, rather than incorporation of pyruvate into the tricarboxylic acid (TCA) cycle for oxidative phosphorylation. Nine of the ten canonical enzymes of glycolysis were represented in the Vn96 captured EVs of both SKBR3 and MCF-7, though only five were observed in MCF-10a. Some elements were exclusively found in the invasive and non-invasive VNEs, such as isoforms of pyruvate kinase and lactate dehydrogenase. Lactate dehydrogenase is perhaps the most widely recognized secreted enzyme of glycolysis contributing to the acidification paradigm of the Warburg effect [36]. Metabolism is thus closely linked to cancer progression, because the ability for a cell to proliferate is dependent upon the availability of nutrients to build new cells. Glycolytic enzymes also protect cancer cells from stress by inhibiting apoptosis, and correlate well with resistance to radio- and chemotherapy [37,38]. Early diversions are promoted toward the pentose phosphate pathway as the means to generate nucleotide and amino acids. Of relevance was the detection of phosphogluconate dehydrogenase in higher abundance for the cancerous cell lines. 

Beyond glycolysis, the provision of raw materials into peripheral biosynthetic pathways is crucial to cancer survival and colonization of areas distal to the primary tumor [39]. For example, glycolysis channels raw material into lipid biosynthesis for membrane expansion and vesicle production [40,41]. Lipogenesis, required for membrane expansion and vesicle production, and typical of the aggressive cancer, would benefit from the donation of precursors. As seen in Table 4, a high proportion of peptides originate from fatty acid synthase (FASN) in the invasive SKBR3. Similarly, tumor protein D52 was also exclusively observed in SKBR3, being implicated in increased capacity for storing lipid typical of invasive cancer cells [42]. The significance of FASN abundance in an invasive phenotype is likely associated with the promotion of membrane biogenesis. FASN is frequently associated with invasive cancer, and has been proposed as a therapeutic target [43]. It is normally expressed in low levels when dietary sources are sufficient. However, FASN expression and activity in cancer cells can be very high, and becomes associated with lipid rafts following cell signaling events [44]. Serum levels of FASN have been found extracellularly in breast cancer [45], and are predictive of colorectal cancer stage [46]. FASN has previously been found in exosomes from multiple cancer cells [32]. Accordingly, it is possible that proportional representation of FASN in exosomes is a prospective biomarker of invasive phenotype [47].

As ligands for the Vn96 affinity peptide, individual canonical heat shock proteins are listed in Table 5. HSP60 was the most abundantly represented heat shock protein among the three cell lines, and most highly expressed in SKBR3. HSP60 is a known surface-displayed molecule, secreted by cancer cells, and an important marker of cancer-derived exosomes [48]. Isoforms of HSP90 were more extensively represented in SKBR3, though also observed in the non-cancerous cell line MCF7. HSP90-alpha constitutes the extracellular isoform, and is particularly characteristic of invasive cancer [49]. Although location of HSP90 isoforms was not determined, this family of proteins is frequently found on the cell surface of cancer cells, and by extension, on derivative vesicles [50]. HSP90 is imperative as a stabilizing chaperone of a broad range of aberrantly overactive receptors and kinases imperative to cancer. Transient HSP–multi protein complexes, coined as the “epichaperome” and found in high prevalence on numerous cancers, have been shown to play important roles in facilitating cell regulation and survival [51]. Thus, while smaller chaperones, HSP10 or HSP27, were observed in higher abundance in the cancerous cell lines, it is unknown if these are independently capable of engaging Vn96. However, HSP10 and 27 have been implicated in multifunctional chaperone networks in invasive breast cancer [52]. HSP chaperone complexes not only present biomarkers for cancer diagnostics, but have been identified as targets for drug therapy [51].

The functional elements of a metastatic cascade reside in secreted proteins invested in HSP-decorated EVs. Cumulatively, these proteins enable cells to detach, invade tissue, and access circulation, while buffering against toxicity. Proteome profiling of the EV materials from malignant vs non-invasive phenotypes reveals multiple features conducive to malignancy, some of which have only been appreciated in the last year. A short selection is provided in Table 6. The role of these proteins in cancer progression is described in the table with reference to literature. For example, protein disulfide isomerases are multifunctional chaperones instrumental in the breakage and rearrangement of disulfide bonds of extracellular matrix proteins, being required for detachment, extravasation, and intravasation at secondary sites, particularly with regard to cellular matrix remodeling. Other proteins may protect or preserve aspects crucial to metabolism (e.g., selenium-binding protein 1), while other proteins may promote expression of specific proteins or enable function to maintain the malignant phenotype (14-3-3 zeta). The Vn96 protocol to isolate EV materials uncovers multiple distinguishing protein features, which collectively constitute candidate biomarkers of breast cancer. 

Previous investigations on breast cancer cell lines [66], or of their secreted exosomes [67], have highlighted proteomic profiles indicative of cancer cell proliferation and mobility, which was also apparent from our study. Our observations also corroborate a proteomic study that identified proteins involved in metabolic and detoxification pathways as highly expressed in HER-2/neu-positive breast cancer [68].

## 4. Conclusions

Vesicles were recovered from extracellular media by peptides with affinity to heat shock proteins, which are abundant on cancer cell surface and derivative vesicles or exosomes. Extracellular vesicles (EVs) were subject to GELFrEE fractionation and proteome analysis via bottom up liquid chromatography mass spectrometry (LC/MS). Enzymes typical of altered metabolic pathways were abundantly represented in EVs from cancer cells. Vesicle-associated proteins from the most invasive phenotype, SKBR3, included most of the canonical enzymes of glycolysis and gluconeogenesis. In contrast, the same proteins were of limited representation or absent in EVs from non-transformed MCF-10a, while MCF-7 yielded an intermediate representation. These observations indicate that the collection of extracellular vesicles from different cancer cell phenotypes may provide insight into the importance of individual enzymes in cancer progression, and further, that the vesicles shed from cancer cells serve as surrogates for profiling abundance of altered metabolic enzymes.

## Figures and Tables

**Table 1:** Top 25 proteins identified in SKBR3 EV fraction, excluding probable contaminants.

SKBR3				
Protein Name	ID	Unique Peptides	PSMs	Function
Enolase	P06733	13	197	metabolism
Fatty acid synthase	P49327	20	139	metabolism
Phosphoglycerate kinase	P00558	11	82	metabolism
Fructose bisphosphatase 1	P09467	11	70	metabolism
GAPDH	E7EUT5	7	64	metabolism
Malate dehydrogenase	E9PDB2	10	59	metabolism
L-lactate dehydrogenase	P00338	8	50	metabolism
Aldehyde dehydrogenase	F8W0A9	8	48	metabolism
Aldolase	H3BPS8	6	33	metabolism
Triosephosphate isomerase	P60174	6	31	metabolism
Glucosidase 2 subunit beta	P14314	6	28	metabolism
Selenium-binding protein 1	Q13228	6	106	binding
60 kDa heat shock protein	P10809	8	101	binding
Protein disulfide-isomerase	I3L2P8	9	63	binding
14-3-3 protein zeta/delta	P63104	6	49	binding
Lamin A/C	Q6UYC3	6	49	binding
Tumor protein D52	D3YTI0	7	44	binding
14-3-3 protein epsilon	P62258	8	38	binding
TER ATPase	P55072	6	25	binding
Cytokeratin 19	P08727	18	635	assembly
Cytokeratin 8	P05787	20	350	assembly
Cytokeratin 18	F8VZY9	8	112	assembly
Cytokeratin 16	P08779	7	109	assembly
alpha-Actinin-4	O43707	11	72	assembly
Myosin-9	P35579	11	26	assembly

**Table 2:** Top 25 proteins identified in MCF-7 EV fraction, excluding probable contaminants.

MCF-7				
Protein Name	ID	Unique Peptides	PSMs	Function
Enolase	P06733	9	103	metabolism
Aldolase	H3BQN4	7	62	metabolism
Fructose bisphosphatase 1	P09467	11	59	metabolism
Triosephosphate isomerase	P60174	8	49	metabolism
Pyruvate kinase	P14618	7	45	metabolism
Phosphoglycerate kinase	B7Z7A9	8	38	metabolism
GAPDH	E7EUT5	6	27	metabolism
Tryptophan-tRNA ligase	P23381	8	23	metabolism
Cathepsin D	P07339	5	20	metabolism
Kynureninase	Q16719	6	15	metabolism
TER ATPase	P55072	5	15	metabolism
Lactoferroxin-C	B7Z4X2	9	14	metabolism
Hexokinase-1	B4DG62	7	13	metabolism
14-3-3 protein zeta/delta	P63104	7	83	binding
14-3-3 protein epsilon	P62258	9	63	binding
Selenium-binding protein 1	Q13228	6	38	binding
HSP 90-a	P07900	5	29	binding
Agrin	O00468	5	25	binding
Protein SET	Q01105	5	21	binding
Cytokeratin 8	P05787	23	557	assembly
Cytokeratin 19	P08727	11	241	assembly
Cytokeratin 18	P05783	13	197	assembly
Actin, cytoplasmic 1	P60709	9	103	assembly
Filamin A	A6NDY9	7	38	assembly
Lamin A/C	Q6UYC3	6	38	assembly

**Table 3:** Top 25 proteins identified in MCF-10a EV fraction, excluding probable contaminants.

MCF-10a				
Protein Name	ID	Unique Peptides	PSMs	Function
Enolase	P06733	5	29	metabolism
GAPDH	E7EUT5	4	15	metabolism
Matrix metalloproteinase-2	B4DWH3	4	7	metabolism
Tripeptidyl-peptidase 1	O14773	3	7	metabolism
Cathepsin D	P07339	3	5	metabolism
Complement component 1r	H0YFH3	3	4	metabolism
Protein disulfide-isomerase	P13667	3	4	metabolism
Anastellin	F8W7G7	12	63	binding
Periostin	F5H628	4	29	binding
Serpin B5	P36952	4	15	binding
TGF-β-induced protein ig-h3	G8JLA8	6	14	binding
Actin, cytoplasmic 1	F5GYT4	4	70	assembly
Laminin subunit gamma-2	F5H430	4	53	assembly
Filamin A	A6NDY9	6	39	assembly
Cytokeratin 8	P05787	4	31	assembly
Alpha-actinin-4	O43707	6	23	assembly
Thrombospondin-1	P07996	5	20	assembly
Laminin subunit gamma-1	P11047	3	16	assembly
alpha-Actinin-1	H9KV75	5	13	assembly
Myosin-9	P35579	4	13	assembly
Tropomyosin a-1 chain	H0YKP3	3	10	assembly
Agrin	O00468	5	9	assembly
Filamin-B	O75369	4	7	assembly
Desmoplakin	P15924	3	4	assembly
Laminin subunit alpha-5	O15230	3	4	assembly

**Table 4:** Summary of proteins involved in metabolic pathways and detected in EV pellets for each cell lines.

Protein Description	Process	Protein Spectral Hits (PSMs)		
		SKBR3	MCF7	MCF10
Hexokinase 1	Glycolysis/Gneo	1	13	0
Glucose-6-phosphate isomerase	Glycolysis/Gneo	1	1	0
Phosphofructokinase	Glycolysis/Gneo	0	0	0
Aldolase (fructose 1,6-bisphosphatase)	Glycolysis/Gneo	138	176	12
Triosephosphate isomerase 1	Glycolysis/Gneo	31	49	2
GAPDH	Glycolysis/Gneo	64	27	15
Phosphoglycerate kinase	Glycolysis/Gneo	82	38	5
Phosphoglycerate mutase	Glycolysis/Gneo	7	3	0
Enolase	Glycolysis/Gneo	197	123	29
Pyruvate kinase	Glycolysis/Gneo	17	45	0
Fatty Acid Synthase	Lipogenesis	139	1	0
Tumor Protein D52	Lipid storage	44	0	0
Lactate dehydrogenase A	Glycolysis/Gneo	50	40	0
Lactate dehydrogenase B	Glycolysis/Gneo	11	0	0
6-phosphogluconate dehydrogenase	Pentose Phosphate	27	22	7
Isocitrate dehydrogenase	TCA	29	14	0

**Table 5:** Canonical heat shock proteins identified in the EV fractions.

Protein Name	SwissProt Accession Number	Peptide Spectral Matches		
		SKBR3	MCF7	MCF10a
HSP10	[B8ZZL8]	7	7	0
HSP60	[P10809]	101	9	0
HSP70-1A/1B	[P08107]	3	7	1
HSP71	[E9PKE3], [E9PQQ4]	0	5	2
HSP27	[P04792], [B4DL87]	32	53	4
GRP78 (Hsp70-5)	P11021	57	16	0
HSP90-alpha	[P07900]	77	29	39
HSP90-beta	[P08238]	70	0	41
GRP94 (Hsp90-B1)	[P14625], [E9PEX3]	12	4	6
	SUM:	347	126	87

**Table 6:** Selected EV pulldown proteins conducive to malignancy, listing peptide spectral matches.

Protein Description	Accession	SKBR3	MCF7	MCF10	Function	Ref.
Selenium-binding protein 1	[Q13228]	106	38	0	Detox	[53]
PDIA1 (P4HB)	[I3L2P8]	63	0	0	Invasion	[54]
PDIA3 (ERP57, GRP58)	[H7BZJ3]	52	6	1	Invasion	[55]
Aldehyde dehydrogenase 1	[F8W0A9]	48	0	0	Detox	[56]
14-3-3 zeta/delta ζ	[P63104]	49	82	0	Enabler	[57]
Sushi Domain Containing D2	[Q9UGT4]	48	0	0	Invasion	[58]
Acylamino-acid-releasing	[P13798]	41	14	0	Detox	[59]
Calcyphosin	[Q13938]	41	0	0	Proliferation	[60]
SS DNA-binding	[Q04837]	38	0	0	Resistance	[61]
Galectin-3-binding	[Q08380]	36	0	0	Invasion	[62]
Calreticulin	[P27797]	33	0	0	Protection	[63]
60S ribosomal P2	[P05387]	31	0	0	Enabler	[64]
Glucosidase 2 beta (80-KH)	[P14314]	28	2	7	Invasion	[65]

## Supplementary Materials

The following are available online at www.mdpi.com/2227-7382/5/4/25/s1, Figure S1: Washes of Vn96 pellet, Figure S2: Gene Ontology, Figure S3: Ingenuity Pathway Analysis, Table S1: Proteins from SKBR3, Table S2: Proteins from MCF-7, Table S3: Proteins from MCF-10a, Table S4: Proteins in ExoCarta database, Table S5: Comparative quantitative analysis by peptide spectral counting.
