# Supplementary material for: Differential Proteome Analysis of Extracellular Vesicles from Breast Cancer Cell Lines by Chaperone Affinity Enrichment
Source: Proteomes. 2017 Oct 8;5(4):25. doi: 10.3390/proteomes5040025 (PMC5748560; doi:10.3390/proteomes5040025)
Supplement: Supplementary file 1 [file proteomes-05-00025-s001.docx]

Article

Differential Proteome Analysis of Extracellular Vesicles from Breast Cancer Cell Lines by Chaperone Affinity Enrichment

Steven G. Griffiths ^1^ Michelle T. Cormier ^2,†^ Aled Clayton ^3^ and Alan A. Doucette ^4,^*

SUPPLEMENTAL FIGURES

TABLE OF CONTENTS:

**Figure S1:** Release of weakly-associated proteins through EB1 and EB2 washes…….. 2

**Figure S2:** Summary from Gene Ontology analysis of total protein content…..……… 3

**Figure S3.** Ingenuity Pathway Analysis………………………………………………….. 4


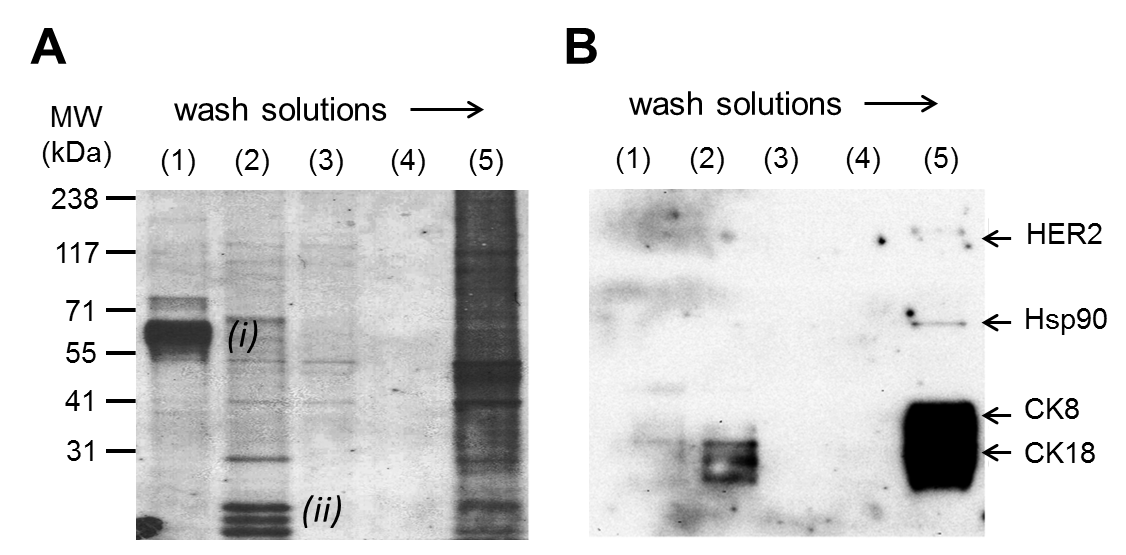


**Figure S1**. Sequential pelleting and resuspension of a Vn96 pull-down with increasingly stringent wash buffers. The total protein stain in (**A**) shows multiple proteins liberated by EB1 (lane 1) and EB2 (lane 2), with molecular weights consistent with albumin *(i)* and histones *(ii)* [1]. EB3 and EB4 buffers (lanes 3, 4) do not extract significant protein. The final EV extraction buffer (USB, lane 5) liberates the bulk of the proteins recovered in the Vn96 pellet. (**B**) A Western blot of Vn96-pelleted material with antibodies to cytokeratin 8/18 is provided. Lanes 1-4 correspond to extractions with buffers EB1-4 respectively, while lane 5 is again the USB extraction buffer. CK8 & 18 are associated with breast cancer cell plasma membrane [2,3] and are serum biomarkers of significance [4]. While marginal CK8/18 is liberated by EB2, the majority required solubilization in USB. The same blot was also probed with antibodies to HSP90 and HER2. Staining is comparably faint due to remaining signal from CK8/18, though bands are consistent with blots shown in Figure 3.

**References**

1. Nangami G, Koumangoye R, Shawn Goodwin J, Sakwe AM, Marshall D, Higginbotham J, Ochieng J. Fetuin-A associates with histones intracellularly and shuttles them to exosomes to promote focal adhesion assembly resulting in rapid adhesion and spreading in breast carcinoma cells. *Exp. Cell. Res.* **2014**, 328, 388-400.
2. Hembrough TA, Li L, Gonias SL. Cell-surface cytokeratin 8 is the major plasminogen receptor on breast cancer cells and is required for the accelerated activation of cell-associated plasminogen by tissue-type plasminogen activator. J Biol Chem. **1996**, *271*, 25684-25691
3. Liu F, Chen Z, Wang J, Shao X, Cui Z, Yang C, Zhu Z, Xiong D. Overexpression of cell surface cytokeratin 8 in multidrug-resistant MCF-7/MX cells enhances cell adhesion to the extracellular matrix. Neoplasia **2008**, *10*, 1275-1284.
4. Ahn SK, Moon HG, Ko E, Kim HS, Shin HC, Kim J, You JM, Han W, Noh DY: Preoperative serum tissue polypeptide-specific antigen is a valuable prognostic marker in breast cancer. Int. J. Cancer **2013**, *132*, 875-881

**
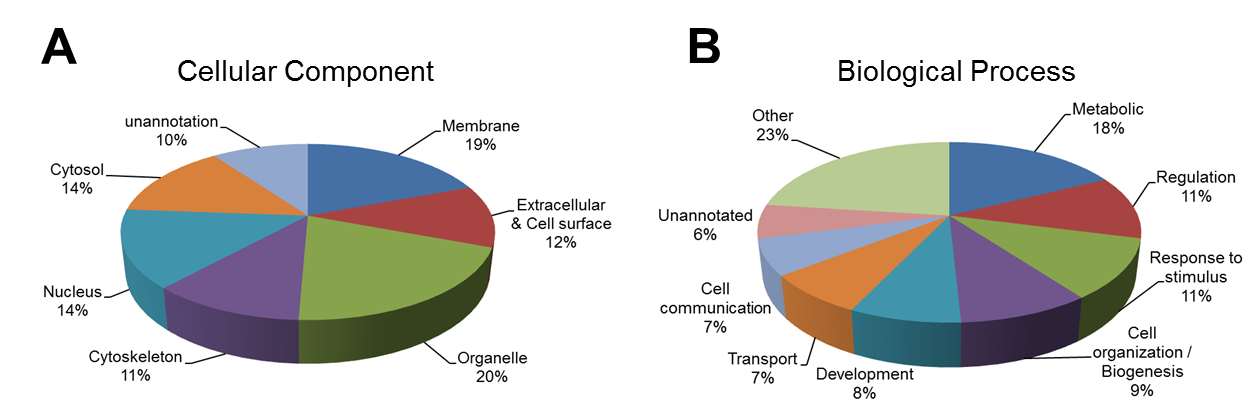
**

**Figure S2.** Gene Ontology distribution of proteins identified in the Vn96 pulldowns according to: (**A**) cellular component; (**B**) molecular function.

**
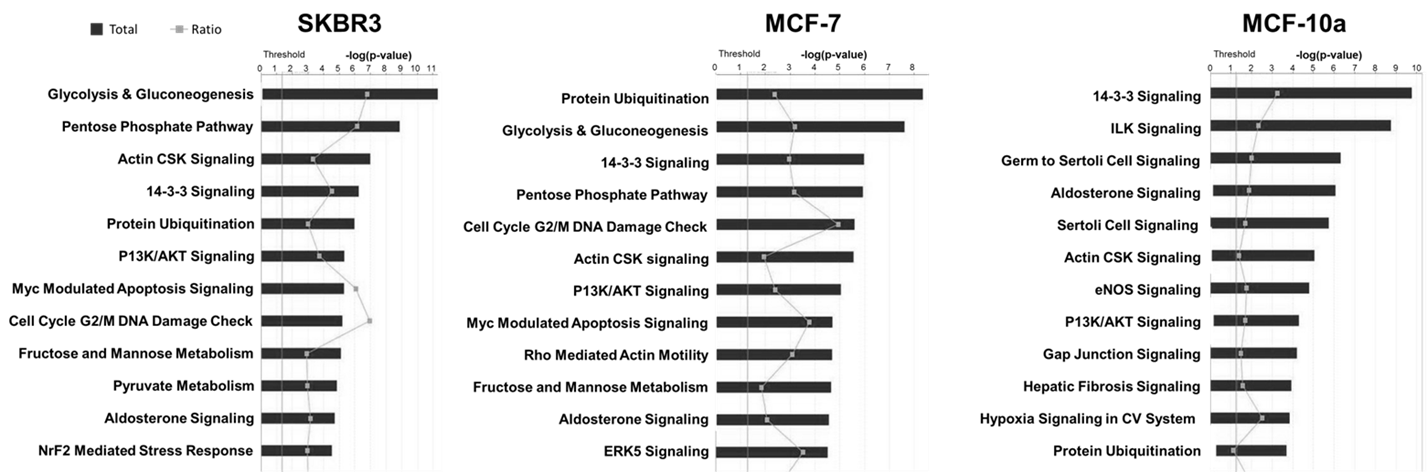
**

**Figure S3.** Ingenuity Pathway Analysis depicting the top 12 canonical pathways represented by the Vn96 EV proteome for SKBR3, MCF-7 and MCF-10a. A –log(p value) above 1.5 signifies that the represented pathway is statistically significant.
